# Long‐Term Outcomes Following Heart Team Revascularization Recommendations in Complex Coronary Artery Disease

**DOI:** 10.1161/JAHA.118.011279

**Published:** 2019-04-04

**Authors:** Tiffany Patterson, Hannah Z.R. McConkey, Fiyyaz Ahmed‐Jushuf, Konstantinos Moschonas, Hanna Nguyen, Grigoris V. Karamasis, Divaka Perera, Brian R. Clapp, James Roxburgh, Christopher Blauth, Christopher P. Young, Simon R. Redwood, Antonis N. Pavlidis

**Affiliations:** ^1^ Division of Cardiovascular The Rayne Institute BHF Centre of Research Excellence King's College London St. Thomas’ Hospital London United Kingdom; ^2^ Department of Cardiothoracic Guy's and St Thomas’ NHS Foundation Trust London United Kingdom; ^3^ Department of Cardiology King's College Hospital NHS Foundation Trust London United Kingdom; ^4^ Department of Cardiology Essex Cardiothoracic Centre Basildon United Kingdom

**Keywords:** coronary artery disease, health outcomes, Heart Team, medication therapy, revascularization, Quality and Outcomes, Mortality/Survival, Cardiovascular Surgery, Percutaneous Coronary Intervention, Revascularization

## Abstract

**Background:**

The Heart Team (HT) comprises integrated interdisciplinary decision making. Current guidelines assign a Class Ic recommendation for an HT approach to complex coronary artery disease (CAD). However, there remains a paucity of data in regard to hard clinical end points. The aim was to determine characteristics and outcomes in patients with complex CAD following HT discussion.

**Methods and Results:**

This observational study was conducted at St Thomas’ Hospital (London, UK). Case mixture included unprotected left main, 2‐vessel (including proximal left anterior descending artery) CAD, 3‐vessel CAD, or anatomical and/or clinical equipoise. HT strategy was defined as optimal medical therapy (OMT) alone, OMT+percutaneous coronary intervention (PCI), or OMT+coronary artery bypass grafting. From April 2012 to 2013, 51 HT meetings were held and 398 cases were discussed. Patients tended to have multivessel CAD (74.1%), high SYNTAX (Synergy between PCI with Taxus and Cardiac Surgery) scores (median, 30; interquartile range, 23–39), and average age 69±11 years. Multinomial logistic regression analysis performed to determine variables associated with HT strategy demonstrated decreased likelihood of undergoing PCI compared with OMT in older patients with chronic kidney disease and peripheral vascular disease. The odds of undergoing coronary artery bypass grafting compared with OMT decreased in the presence of cardiogenic shock and left ventricular dysfunction and increased in younger patients with 3‐vessel CAD. Three‐year survival was 60.8% (84 of 137) in the OMT cohort, 84.3% (107 of 127) in the OMT+PCI cohort, and 90.2% in the OMT+coronary artery bypass grafting cohort (92 of 102).

**Conclusions:**

In our experience, the HT approach involved a careful selection process resulting in appropriate patient‐specific decision making and good long‐term outcomes in patients with complex CAD.

**Clinical Perspective**

**What Is New?**

- There remains a paucity of data on Heart Team recommendations and outcomes; this observational study describes the factors influencing decision making and long‐term outcomes in patients with complex coronary artery following Heart Team discussion and implementation.

**What Are the Clinical Implications?**

- The Heart Team approach comprised a careful selection process resulting in appropriate patient‐specific decision making with good clinical outcomes in patients with complex coronary artery disease; we therefore believe that, in patients in whom there is anatomical or clinical equipoise, the Heart Team approach should be the emerging default strategy for complex decision making.

## Introduction

Ad‐hoc percutaneous coronary intervention (PCI) has been proven safe and effective when compared with delayed PCI in observational studies.^1^ However, ad‐hoc PCI may not be appropriate in the presence of complex coronary artery disease (CAD). In such situations, revascularization strategies can be unclear; thus, involvement of the Heart Team (HT) can maximize the potential for multidisciplinary input, thus providing the patient with sufficient information to support informed decision making.^2^, ^3^ Since its inception just over a decade ago, the HT has evolved substantially.^3^ The HT concept comprises integrated, active decision making between cardiologists and cardiac surgeons. Patients with complex CAD referred for revascularization strategy following HT discussion and consensus may fare better than those selected for a particular strategy in its absence.^2^, ^3^, ^4^ Current guidelines assign a Class I recommendation (level of evidence C) for implementation of an HT approach to complex CAD and Class IIa recommendation for calculation of SYNTAX (Synergy between PCI with Taxus and Cardiac Surgery) scores (level of evidence B) to aid decision making.^2^

The advantages to HT decision making have been previously described, but broadly include input from physicians of differing backgrounds to facilitate complex and patient‐specific decision making in a timely and nonemergent setting.^5^, ^6^ However, barriers also exist to implementation of the HT approach.^5^, ^6^, ^7^ Perhaps the most significant of these is balancing multiple opinions and generating a final decision in a systematic manner and communicating this with the patient, particularly in urgent clinical scenarios.^6^, ^7^ The HT is still an emerging concept in cardiovascular medicine as compared with other specialities, and for various reasons, there is still considerable variability in the care delivered to patients with complex CAD.^8^, ^9^ The HT concept is still not yet widely adopted, and questions remain about the feasibility and efficacy of the HT approach.

Despite these concerns, we believe the HT concept to have a fundamental role in integrating evidence‐based medicine and a multidisciplinary approach to decision making among an aging demographic and the rapidly evolving field of cardiovascular medicine. We have previously reported implementation and consistency of an HT approach in a single, large academic center.^7^ However, there remains a paucity of data in regard to hard clinical end points following HT discussion. Thus, the primary aim of this observational study was to describe the factors influencing decision making and long‐term outcomes in patients with complex CAD following HT discussion and implementation.

## Methods

The data, analytical methods, and study materials will not be made available to other researchers for purposes of reproducing the results or replicating the procedure without previous approval from the National Health Service Health Research Authority. Data collection within the National Health Service is performed without explicit consent for provision of healthcare, administrative, and clinical audit purposes (local or national) and is performed under the auspices of European Law—General Data Protection Regulation (GDPR) Act (May 2018): article 6(1)(e); 9(2)(h); special category 9(2)(i). These data were extracted retrospectively for assessment of healthcare quality, delivery, and outcomes, and therefore separate institutional review board approval was not required for this work.

### Study Design and Population

This observational study was conducted at a single center: St Thomas’ Hospital (London, UK). In our institution, HT meetings with a specific focus on CAD were conducted on a weekly basis. Case referral comprised patients aged >18 years with CAD; pediatric and isolated structural cases were not included, and these were discussed in separate, dedicated HT meetings. Case referrals included internal and external referrals (up to 5 affiliated district general hospitals) to cardiologists and/or cardiac surgeons or direct referrals to the HT meeting. Case mixture included unprotected left main CAD, 3‐vessel CAD, 2‐vessel CAD including proximal left anterior descending artery, and CAD whereby the referring physician believed there to be either anatomical and/or clinical equipoise with regard to revascularization strategy. Case collection, presentation, and documentation of recommended mode and extent of revascularization were performed by a dedicated HT coordinator. Attendance at each meeting required a minimum of 3 physicians: an interventional cardiologist, a cardiac surgeon, and a noninvasive cardiologist. Additional relevant clinical data, functional status, and patient characteristics were incorporated in the final decision making when determining optimal mode of treatment. Meetings comprised open discussions using the latest evidence‐based management strategies.

HT outcome was classed as medical therapy, PCI, or surgical intervention. SYNTAX scores were calculated using the online tutorial and calculator to minimize variability. The SYNTAX score has been previously validated as an appropriate predictor of adverse events and a useful tool to aid treatment allocation (PCI or coronary artery bypass graft surgery [CABG]).^2^, ^3^ Left ventricular (LV) dysfunction was classified as an ejection fraction below 50%. Canadian Cardiac Society class was used to quantify the level of angina; New York Heart Association (NYHA) class was used to quantify the level of dyspnea. Patients were categorized as unstable if the clinical picture had triggered an acute hospital admission. Acute coronary syndrome (ACS) was categorized as ST‐segment elevation myocardial infarction or non‐ST‐segment elevation myocardial infarction based on biomarker and electrocardiographic criteria.^10^ In‐hospital mortality was defined as patient death in‐hospital. Life status tracking was achieved in 100%, through linkage with the Office of National Statistics, thus represents in‐hospital mortality and subsequent mortality following discharge.

### Statistical Analysis

Categorical data are presented as counts and percentages, and comparison between groups performed using chi‐square test; continuous data of normal distribution are presented as mean±SD, and analysis was performed using 1‐way ANOVA; data that were not of normal distribution are presented as median values (interquartile range). The variance inflation factor was used to determine colinearity using standardized cutoffs. In patients with complex CAD following HT discussion and implementation, 30‐day, 1‐, and 3‐year outcomes in addition to the distribution of baseline characteristics (age, sex, body mass index, previous myocardial infarction, previous PCI, previous CABG, diabetes mellitus, hypertension, hypercholesterolemia, smoking history, chronic kidney disease [CKD], peripheral vascular disease (PVD), LV dysfunction, extent of coronary disease (including left main stem disease), unstable presentation, cardiogenic shock, SYNTAX score, Canadian Cardiac Society, and NYHA class were examined. Significant colinearity was demonstrated between NYHA class and a large number of covariates; therefore, NYHA class was not included in the final analysis.

To determine the odds of undergoing optimal medical therapy (OMT), PCI, or CABG as the preferred HT strategy, multivariable, multinomial logistic regression models were developed with “OMT” as the reference outcome group. Adjusted odds ratios (ORs) with 95% CIs are reported. To determine independent predictors for 30‐day and 1‐ and 3‐year survival, multivariable logistic regression models were used to generate adjusted ORs. To limit the number of variables for the final multivariable models, forward step‐wise regression was performed using the above covariates (entry criteria, *P*<0.05; exit criteria, *P*>0.1); only significant variables were used in the final model. Final model selection was performed using multiple imputation (Fully Conditional Specification, SPSS v24.0; IBM Corp., Armonk, NY) to impute missing data on baseline covariates by chained equations to create 5 multiply imputed data sets to maximize statistical power. The variables used in the final model for the 3‐level categories of HT decision were age, sex, previous myocardial infarction, previous PCI, previous CABG, diabetes mellitus, hypertension, hypercholesterolemia, extent of coronary disease, syntax group, LV dysfunction, body mass index, smoking history, PVD, CKD, and ACS. The variables used in the final model for the odds of 30‐day and 1‐ and 3‐year survival were CKD, age, extent of coronary disease, PVD, CKD, smoking history, and previous PCI.

Time to event analysis was performed using Kaplan–Meier curves. To handle participant crossover from initial HT recommendation to final strategy, in addition to the intention to treat analysis, an additional as‐treated sensitivity analysis was performed for clinical outcome data. Cox proportional hazard analyses model was performed to generate hazard ratios (HRs) for HT strategies using the following variables: CKD, age, extent of coronary disease, PVD, CKD, smoking history, and previous PCI. All *P* values were 2‐sided with a significance threshold *P*<0.05. Statistical analysis was performed using SPSS software (v24.0; IBM Corp.).

## Results

### Patient Identification and HT Meeting Characteristics

**Figure 1:**
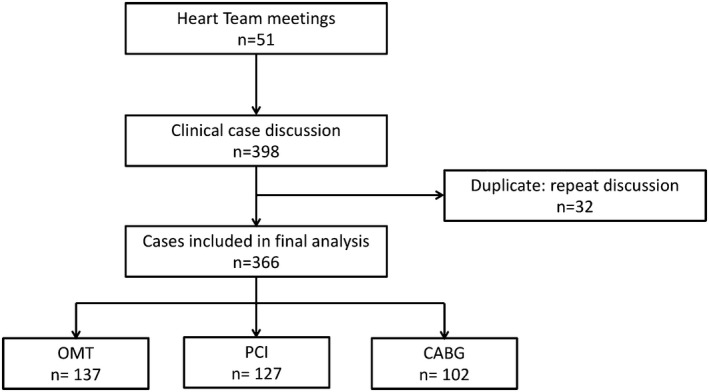
Flow diagram demonstrating case identification following 51 Heart Team meetings between April 2012 and April 2013. CABG indicates coronary artery bypass grafting; OMT, optimal medical therapy, PCI, percutaneous coronary intervention.

Figure 1 details case identification. From April 2012 to April 2013, 51 HT meetings were held and a total of 398 cases were discussed. The meetings were attended by a median of 3 interventional cardiologists, 1 noninterventional cardiologist, and 2 cardiac surgeons. Of the 398 cases discussed, 32 cases were repeat discussion (8.0%) following the outcome of further investigations; therefore, 366 of the 398 (92.0%) cases were analyzed. Initial HT recommendation was for CABG in 26.2% (95 of 366) of cases, PCI in 17.8% (65 of 366) of cases, OMT in 33.1% (121 of 366) of cases, and further investigation in 23.2% (85 of 366) of cases. The final HT recommendation and outcome was for CABG in 27.9% (102 of 366) of cases, PCI in 34.7% (127 of 366) of cases, and OMT in 37.4% (137 of 366) of cases. Table 1 displays baseline characteristics, including cardiovascular risk factors, extent of coronary disease (1‐, 2‐, or 3‐vessel), SYNTAX scores, and clinical presentation in the overall cohort and according to final allocated treatment strategy.

**Table 1:** Patient Demographics of the Cohort of Patients Identified From the 51 Heart Team Meetings Conducted Between April 2012 and April 2013

Demographics	Overall, n=366	PCI, n=127	CABG, n=102	OMT, n=137	*P* Value
Age, y	69±11	68±10	66±10	72±11	0.002
Sex, male	310 (84.7)	92 (72.4)	87 (85.3)	131 (95.6)	0.077
BMI, kg/m^2^	28±6	28±5	28±6	28±6	0.092
Previous MI	171 (46.7)	58 (45.7)	62 (60.8)	51 (37.2)	0.005
Previous PCI	74 (20.2)	35 (27.6)	20 (19.6)	19 (13.9)	0.218
Previous CABG	35 (9.6)	17 (13.4)	6 (5.9)	12 (8.8)	0.255
Diabetes mellitus	110 (30.1)	43 (33.9)	27 (26.5)	40 (29.2)	0.723
Hypertension	247 (67.5)	96 (75.6)	82 (80.4)	69 (50.4)	0.298
Cholesterol^a^	192 (52.5)	108 (85.0)	···	82 (59.9)	0.742
Smoking history	186 (50.8)	67 (52.8)	72 (70.6)	47 (34.3)	0.191
Chronic kidney disease	30 (8.2)	7 (5.5)	3 (2.9)	20 (14.6)	0.644
PVD	16 (4.4)	4 (3.1)	7 (6.9)	5 (3.6)	0.500
LV dysfunction (EF<50%)	125 (34.2)	22 (17.3)	40 (39.2)	63 (46.0)	0.017
LMS disease (>50%)	86 (23.5)	29 (22.8)	30 (29.4)	27 (19.7)	0.693
Coronary disease >50%^b^					
1 vessel	35 (9.6)	12 (9.4)	4 (3.9)	19 (13.9)	0.064
2 vessels	91 (24.9)	42 (33.1)	21 (20.6)	28 (20.4)	0.220
3 vessels	180 (49.2)	53 (41.7)	73 (71.6)	54 (39.4)	0.027
SYNTAX score	30 (23–39)	29 (21–37)	31 (25–39)	31 (23–40)	0.149
Logistic EuroScore	4.9 (2.7–9.9)	6.2 (2.9–12.5)	3.9 (1.9–6.5)	6.4 (12.9–12.7)	0.003
Clinical presentation					
ACS/unstable angina	145 (39.6)	53 (41.7)	43 (42.2)	49 (35.8)	0.296
Cardiogenic shock	13 (3.6)	6 (4.7)	1 (1.0)	6 (4.4)	0.336
CCS class					
1 to 2	145 (39.6)	42 (33.1)	42 (41.2)	61 (44.5)	0.274
3 to 4	144 (39.3)	50 (39.4)	47 (46.1)	47 (34.3)	0.049
NHYA					
1 to 2	197 (53.8)	75 (59.1)	77 (75.5)	45 (32.8)	0.003
3 to 4	115 (31.4)	33 (26.0)	22 (21.6)	60 (43.8)	0.003

Demographics are reported in the overall population and according to treatment arm. Unadjusted *P* values are displayed and used to compare characteristics between the treatment arms. Categorical data are described as n (%), where n is the total number of patients identified as fulfilling the specified demographic and (%) is the percentage. Continuous data are described as mean±SD; data that are not normally distributed are described as median (interquartile range). ACS indicates acute coronary syndrome; BMI, body mass index; CABG, coronary artery bypass grafting; CCS, Canadian Cardiac Society; EF, ejection fraction; LMS, left main stem; LV, left ventricle; MI, myocardial infarction; NYHA, New York Heart Association; OMT, optimal medical therapy; PCI, percutaneous coronary intervention; PVD, peripheral vascular disease; SYNTAX, Synergy between PCI with Taxus and Cardiac Surgery.

a Data on hypercholesterolemia were not available in the surgical cohort.

b Coronary disease not including left main stem disease.

### HT Patient Demographics

In the overall cohort of patients discussed by the HT, 84.7% of patients were male, with an average age of 69±11 years. Nearly half (49.2%) of the patients had 3‐vessel coronary disease, with median syntax score of 30 (interquartile range, 23–39) and mean LV ejection fraction of 48±14%. In the overall cohort of patients discussed by the HT, 39.1% (145 of 366) had unstable symptoms presenting with an ACS. Patients in whom the final decision was CABG were more likely to have had a previous myocardial infarction (unadjusted *P*=0.005), impaired LV function (unadjusted *P*=0.017), 3‐vessel coronary disease (unadjusted *P*=0.027), and a lower EuroScore (*P*=0.003). Conversely, patients who were selected by the HT to undergo PCI were more likely to be older (unadjusted *P*=0.002), with significant/unstable angina (Canadian Cardiac Society class III–IV; unadjusted *P*=0.049). Patients who were selected for medical therapy were more likely to have shortness of breath as the predominant symptom (NYHA III–IV; unadjusted *P*=0.003).

**Figure 2:**
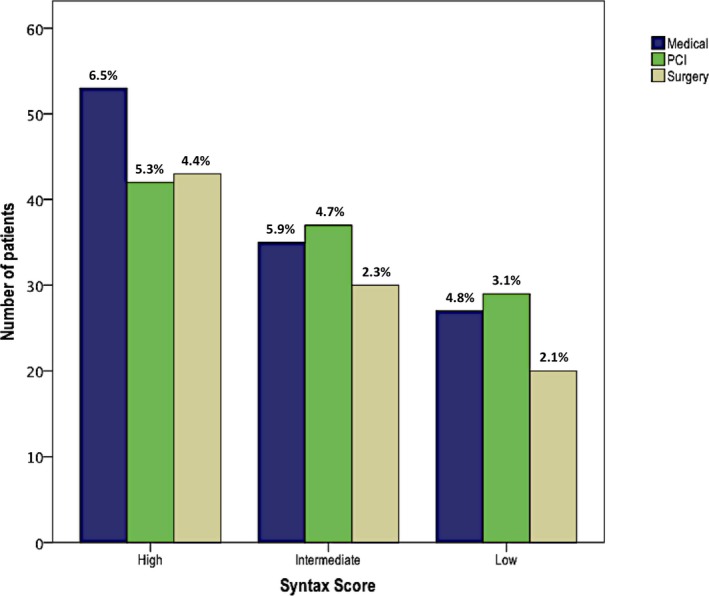
Heart Team approach stratified by low, medium, or high syntax score in patients treated with optimal medical therapy (OMT), OMT+percutaneous coronary intervention (PCI), and OMT+coronary artery bypass grafting in the 366 patients discussed over the 1‐year period. Median EuroScore of each subgroup is depicted as a percentage (%) on the bar chart.

HT recommendation according to high, intermediate, or low SYNTAX score is depicted graphically in Figure 2. In the cohort of patients categorized as high SYNTAX score (>33), a numerically higher number of cases were treated with OMT; however, this did not reach statistical significance. Furthermore, there was no significant difference in overall HT decision making with regard to OMT (*P*=0.862), PCI (*P*=0.529), or CABG (*P*=0.744) when patients were stratified according to a low, medium, or high SYNTAX score.

### Preferred HT Strategy

Multivariable, multinomial logistic regression analysis was performed in patients with complex CAD following HT discussion and implementation to determine the baseline characteristics associated with a preferred HT recommendation (Table 2). This demonstrated that with increasing age, patients were less likely to receive PCI (OR, 0.96; 95% CI, 0.84–0.99; *P*=0.003) or CABG (OR, 0.95; 95% CI, 0.93–0.97; *P*<0.001) compared with medical therapy. Patients who had undergone previous CABG were less likely to undergo redo CABG compared with medical therapy (OR, 0.3; 95% CI, 0.1–0.7; *P*=0.008). Patients with a smoking history (OR, 2.2; 95% CI, 1.1–4.5; *P*=0.02) or hypertension (OR, 2.3; 95% CI, 1.2–4.4; *P*=0.012) were more likely to undergo CABG compared with medical therapy. In the presence of CKD, patients were less likely to receive PCI (OR, 0.2; 95% CI, 0.07–0.48; *P*=0.001) or CABG (OR, 0.11; 95% CI, 0.02–0.46; *P*=0.004) compared with medical therapy. In the presence of PVD, patients were less likely to receive PCI (OR, 0.24; 95% CI, 0.07–0.84; *P*=0.027) compared with medical therapy. In the presence of cardiogenic shock (OR, 0.15; 95% CI, 0.03–0.77; *P*=0.024) or LV dysfunction (OR, 0.38; 95% CI, 0.19–0.75; *P*=0.007), patients were less likely to receive CABG compared with medical therapy. Patients with single‐vessel coronary disease (OR, 0.142; 95% CI, 0.04–0.56; *P*=0.007) were less likely than patients with 3‐vessel coronary disease to receive CABG compared with medical therapy.

**Table 2:** Multivariable Multinomial Logistic Regression Analysis Demonstrating Significant Associations Between Covariates (Baseline Characteristic) and Selected HT Strategy With Medical Therapy as the Reference Category

Variable	PCI	*P* Value	Surgery	*P* Value
Age, y	0.96 (0.84–0.99)	0.003	0.95 (0.93–0.97)	<0.001
Previous CABG	0.60 (0.30–1.30)	0.202	0.30 (0.10–0.70)	0.008
Hypertension	1.60 (0.90–2.90)	0.100	2.30 (1.20–4.40)	0.012
Smoking	1.30 (0.70–2.40)	0.380	2.20 (1.10–4.50)	0.020
Chronic kidney disease	0.20 (0.07–0.48)	0.001	0.11 (0.02–0.46)	0.004
Peripheral vascular disease	0.24 (0.07–0.84)	0.027	0.54 (0.18–1.64)	0.268
Cardiogenic shock	0.80 (0.31–2.07)	0.638	0.15 (0.03–0.77)	0.024
LV dysfunction	0.60 (0.31–1.17)	0.131	0.38 (0.19–0.75)	0.007
Coronary disease				
1 vessel	0.64 (0.24–1.60)	0.340	0.14 (0.04–0.56)	0.007
2 vessel	1.40 (0.76–2.70)	0.260	0.50 (0.25–1.01)	0.054
3 vessel^a^	…		…	

Data are presented as adjusted odds ratios (95% CIs). CABG indicates coronary artery bypass grafting; LV, left ventricular; PCI, percutaneous coronary intervention; HT, Heart Team.

a Reference group.

### HT Clinical Outcomes

Multivariable logistic regression analysis was performed in patients with complex CAD following HT discussion and implementation to determine independent risk factors (ORs) for 1‐ and 3‐year survival (Table 3). Factors negatively associated with 1‐year survival were increased age (OR, 0.862; 95% CI, 0.799–0.929; *P*<0.001) and CKD (OR, 0.052; 95% CI, 0.011–0.243; *P*<0.001). Factors negatively associated with 3‐year survival were the same as those for 1‐year survival. Absence of smoking history was positively associated with 3‐year survival (OR, 3.236; 95% CI, 1.331–7.871; *P*=0.010).

**Table 3:** Multivariable Logistic Regression Analysis Demonstrating Significant Associations Between Covariates and (1) 30‐Day, (2) 1‐Year, and (3) 3‐Year Survival

Variable	Adjusted Odds Ratio	95% CI	*P* Value
30‐d survival			
Peripheral vascular disease (Absence)	25.11	1.45 to 434.44	0.027
1‐y survival			
Age	0.86	0.80 to 0.93	<0.001
Chronic kidney disease	0.05	0.01 to 0.24	<0.001
3‐y survival			
Age	0.88	0.83 to 0.93	<0.001
Previous PCI	0.37	0.14 to 1.01	0.051
Smoking history (absence)	3.24	1.33 to 7.87	0.010
Chronic kidney disease	0.08	0.02 to 0.34	0.001

PCI indicates percutaneous coronary intervention.

**Figure 3:**
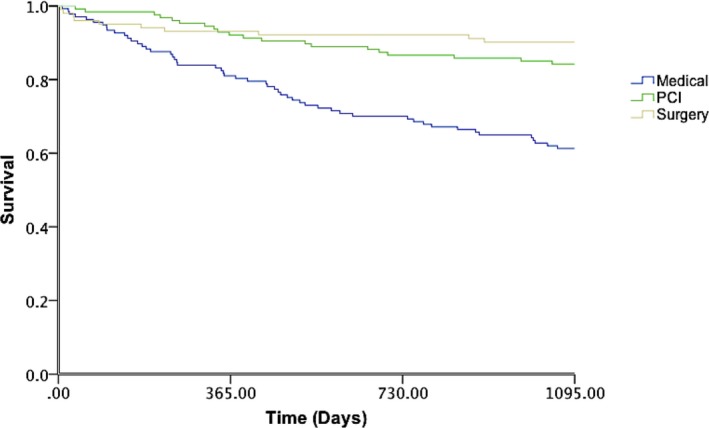
Kaplan–Meier survival curves demonstrating survival in each of the 3 HT strategies (OMT, OMT+PCI, and OMT+CABG) over the 3‐year period. Medical therapy was associated with a 4.5‐fold increased risk of mortality compared with CABG and PCI (HR, 4.588; 95% CI, 2.333–9.021; *P*<0.001). CABG indicates coronary artery bypass grafting; HR, hazard ratio; OMT, optimal medical therapy, PCI, percutaneous coronary intervention.

In the overall cohort of patients with complex CAD following HT discussion and implementation, survival was 98.6% (361 of 366) at 30 days, 88.3% (323 of 366) at 1 year, and 77.3% (283 of 366) at 3 years. Kaplan–Meier curves were generated to 3 years for the 3 treatment arms (Figure 3). Survival at 30 days stratified according to treatment arm was 97.8% (134 of 137) in the OMT cohort, 100% (127 of 127) in the PCI cohort, and 98.2% in the CABG cohort (100 of 102). Survival at 1 year stratified according to treatment arm was 81.0% in the OMT cohort, 92.1% in the PCI cohort, and 93.1% in the CABG cohort. Survival at 3 years was 60.8% (84 of 137) in the OMT cohort, 84.3% (107 of 127) in the PCI cohort, and 90.2% in the CABG cohort (92 of 102). Cox proportional hazards model was used to generate HRs to 3 years for preferred HT strategy. This demonstrated that the risk of mortality was greater in the cohort of patients selected to receive OMT arm compared with the cohort selected to receive CABG and PCI (HR, 4.588; 95% CI, 2.333–9.021; *P*<0.001). There was no significant difference in clinical outcomes at 3 years between the intention‐to‐treat and as‐treated analysis, suggesting that the original analysis was robust to sensitivity analysis (HR, 3.849; 95% CI, 2.012–7.363; *P*<0.001). Subgroup analysis of the cohort of patients with complex CAD discussed by the HT that underwent revascularization was performed to generate HRs to 3 years. This demonstrated no significant difference in mortality at 3 years between CABG and PCI (*P*=0.217).

## Discussion

In this 3‐year prospective, observational study that examined patients with complex CAD that underwent HT discussion and implementation, the main findings were as follows: (1) The demographic of patients discussed had a high SYNTAX score, LV impairment with a mean age of 69; (2) patients had increased odds of receiving PCI if they were in cardiogenic shock or had 3‐vessel coronary disease not including left main stem; (3) patients had increased odds of receiving surgery if they were younger or had isolated left main stem disease; (4) there were increased odds of receiving OMT in isolation with increasing age and coexisting diabetes mellitus; and (5) there was no difference in survival between CABG and PCI when patients underwent HT discussion and implementation of either revascularization strategy in this high‐risk cohort.

In our experience, the HT approach was effective in generating appropriate patient‐specific decision making in patients with complex CAD. The cohort of patients discussed in our HT meetings aligned with American Heart Association recommendations, and the majority of patients tended to have a high syntax score (>33) and were of older age with significant risk factors for coronary disease. A significant challenge to HT implementation is the management of patients who require urgent decision making; this is likely to explain why only 4% of patients discussed presented in cardiogenic shock. In our institution, where necessary, such patients are stabilized with circulatory and ventilatory support until delivery of definitive care plan. Interestingly, a high proportion of patients had presented with unstable symptoms or ACS (39%), despite the need for urgent clinical decision making in such patients; this may suggest a growing familiarity with the HT approach and therefore an increased tendency for physicians to use the HT as a multidisciplinary forum to discuss inpatient care.

We found that patients selected for a strategy of medical therapy tended to be older. The reasoning behind this could be 2‐fold: First, revascularization in this cohort is less likely to carry prognostic benefit. Second, with increasing age, it may be that patients tend to prefer a less‐invasive approach to symptom management. Furthermore, it may reflect unmeasured confounders that are not captured in traditional surgical and PCI databases, including frailty and reduced mobility. Interestingly, diabetes mellitus was also associated with an increased tendency for medical therapy; this is likely to reflect the diffuse coronary disease and lack of interventional targets associated with long‐standing diabetes mellitus rendering revascularization futile in such a cohort. Patients who were selected for medical therapy were more likely to have shortness of breath as the predominant symptom; it may be that such patients had multiple possible causes for breathlessness, and in such patients, in the absence of angina, revascularization may not have been thought to be of symptomatic or prognostic benefit.

Patients selected for CABG tended to be younger, reflecting the current data, which support better long‐term outcomes associated with CABG. Interestingly, however, as the extent of coronary disease increased, patients were less likely to receive CABG and more likely to receive PCI. The cohort of patients discussed in the HT represents those with complex CAD and is not representative of the overall population of patients presenting with coronary disease; therefore (based on our HT patient demographics), this is most likely attributable to the presence of diffuse disease and lack of interventional targets in an older age group, with multiple risk factors often being offered treatment for symptomatic benefit.

Patient survival was recorded from the time of HT discussion; this would potentially explain the excellent 30‐day outcomes in these patients. This could also be attributable to self‐selection and survival to HT discussion. We demonstrated improved clinical outcomes associated with PCI and CABG when compared with medical therapy in our cohort of HT patients, although recent trials have shown no difference between OMT and PCI with respect to long‐term outcomes. This was despite there being a numerically higher number of ACS and unstable patients in the revascularization groups compared with the medical therapy arm. Importantly, these data do not demonstrate OMT to be an inferior strategy. The increased mortality in the OMT arm is likely attributable to factors that were not captured by the database, but taken into consideration by the HT including frailty and comorbidities not captured within the cardiovascular database. In addition, public reporting of outcomes may have led to risk avoidance in this cohort of patients; thus, patients who were not intervened on may have been perceived to be too high risk.

Similar to previous studies, there was an early mortality associated with CABG, likely attributable to perioperative risk; thereafter, CABG was associated with long‐term outcomes that were numerically superior to other HT strategies. Although there was no significant difference in 3‐year survival between PCI and CABG, this cohort of high‐risk patients with complex CAD represent a select group in whom measured decision making, HT discussion, and implementation lead to enhanced overall survival. It is possible that patient selection for revascularization tended to favor those who are most likely to have the best long‐term outcomes. However, patients undergoing HT discussion are unarguably a complex cohort of patients, often with multiple comorbidities, and it is not possible to derive causality from such observational data given that there are multiple confounders not captured by traditional risk scoring. In this cohort of patients, we have shown the HT to be effective. As familiarity grows with the HT approach, we hope that there will be an increased tendency for physicians to use the HT as the default decision making strategy for patients with complex CAD.

## Limitations

There are a number of limitations in this study that are inherent to observational studies that preclude conclusions with regard to causality. HT meetings comprise only a small proportion of patients presenting with coronary disease, thus representing perhaps the highest‐risk cohort in which there may be clinical or anatomical equipoise, and therefore outcomes are not representative of the wider population. The assessment of clinical equipoise was subjective and dependent on the referring physician. The cohort is not representative of the overall population of patients presenting with coronary disease, and therefore findings are not transferrable to the general population. There was no control group comparator in this study; we therefore cannot determine whether the patients fared better with a HT team approach. Furthermore, we did not include patient preference in this study or functional status; these would have been useful when objectively assessing HT decision making. Syntax scores have previously been shown to be subject to observer variation, as such for the purposes of analysis scoring was divided into tertiles, which has been shown to have a better level of agreement.^11^

## Conclusion

This observational study reports 3‐year clinical outcomes in patients with complex CAD that underwent HT discussion and implementation. In our experience, the HT approach involved a careful selection process resulting in appropriate patient‐specific decision making with good clinical outcomes in patients with complex CAD. We therefore believe that, in patients in whom there is anatomical or clinical equipoise, the HT approach should be the emerging default strategy for complex decision making.

## Sources of Funding

This study was supported by British Heart Foundation Clinical Research Training Fellowships: Patterson (FS/14/11/30526), McConkey (FS/16/51/32365), and a National Institute for Health Research Academic Clinical Lectureship: Patterson.

## Disclosures

None.
